# Sekundäre maligne Neoplasien auf dem Boden eines Lynch-Syndroms mit koinzidenter Colitis ulcerosa

**DOI:** 10.1007/s00104-025-02251-w

**Published:** 2025-03-03

**Authors:** Anne Kristin Fischer, Anton Kroesen, Reinhard Büttner

**Affiliations:** 1 Institut für Pathologie, Universität zu Köln, Kerpener Str. 62, 50937 Köln, Deutschland; 2 Krankenhaus Porz am Rhein, Urbacher Weg 19, 51149 Köln, Deutschland

## Anamnese

Berichtet wird über eine 26-jährige Patientin mit Colitis ulcerosa (CU) und bekannter familiärer Vorbelastung durch ein Lynch-Syndrom. Im Rahmen einer Kontrollkoloskopie fanden sich erstmals zwei suspekte Schleimhautareale sowohl im Colon ascendens nahe der rechten Kolonflexur als auch im Colon descendens. CT-morphologisch erhärtete sich der Verdacht auf zwei invasive Kolonkarzinome. Die Probebiopsien bestätigten die Diagnose. Daraufhin erfolgte eine komplikationslose Ileokoloproktomukosektomie.

## Pathologischer Befund

**Abb. 1:**
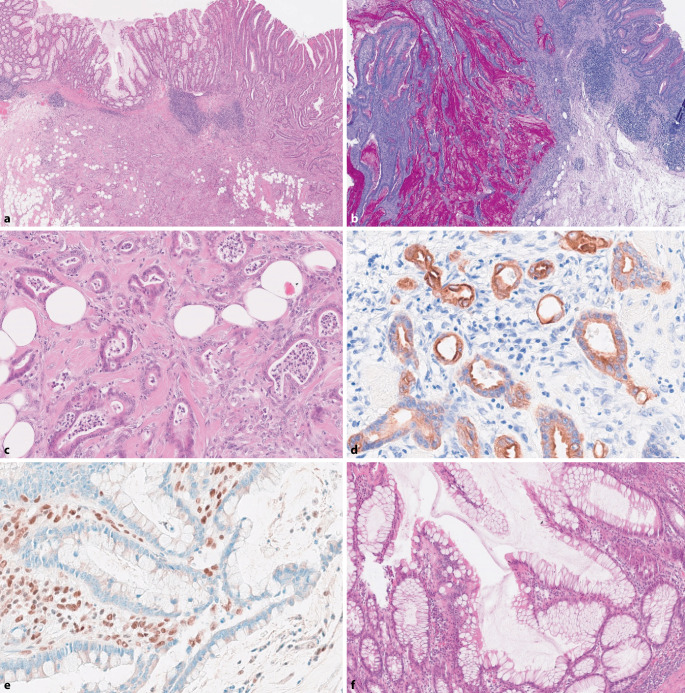
**a** HE-Übersicht des fortgeschrittenen Adenokarzinoms im Colon descendens mit heterogenem Aspekt und nur geringer Immunreaktion. **b** PAS-Übersicht des Adenokarzinoms im Colon ascendens mit Darstellung der rundlichen Tumorformation, umgeben von einem stark ausgeprägten Lymphozytenwall, mit weit über 50 % Muzinsubstanzen und hierin teils flottierenden, zytologisch nur geringe Atypien aufweisenden Tumorzellen. **c** Subserosainfiltration der Tumorzellen, morphologisch teils an ein duktales Pankreaskarzinom erinnernd, teils mit prominenter hyaliner Sklerose des desmoplastisch transformierten Hintergrundstromas und in diesen Abschnitten (**d**) kräftiger CK7-Positivität. **e** Verlust der nukleären MSH2-Expression in den Tumorzellkernen, Lymphozyten im Stroma zeigen als interne Kontrolle eine erhaltene nukleäre Expression. **f** Dysplasiefreie Kolonschleimhaut mit deutlich architekturgestörten, basal ramifizierenden Krypten mit Becherzellhyperplasie

Das Ileokoloproktokolektomiepräparat mit einem 1,5 cm langen terminalen Ileum-, einem 61 cm langen Kolon- und einem 6,5 cm langen Rektumanteil zeigte zwei jeweils 1,9 cm und 1,4 cm messende ulzerierte Schleimhautläsionen mit makroskopisch infiltrativem Aspekt zumindest bis in die Muscularis propria. Histologisch konnten in beiden Lokalisationen zwei von der Kolonschleimhaut ausgehende Karzinome nachgewiesen werden. Der Tumor im Colon descendens wies ein tubuloalveolär-drüsiges Muster mit begleitender Stromadesmoplasie auf. Die einzelnen Tumordrüsen waren von einem einreihigen kubischen bis mehrreihigen zylindrischen Epithel ausgekleidet. Damit erinnerten die Tumorinfiltrate morphologisch an ein duktales Adenokarzinom des Pankreas und exprimierten demgemäß auch CK7 (Abb. 1d) und CA19.9. Anderenorts zeigte sich ein für kolorektale Karzinome (CRC) typisches vernetztdrüsiges Muster mit mehrreihiger zylinderepithelialer Auskleidung. Das Tumorgewebe war gering lymphozytär infiltriert. Bei Ausbreitung in das subserosale Fettgewebe und fokaler Lymphangio- und Hämangioinvasion sowie einer Lymphknotenmetastase ohne extrakapsuläre Extension folgte als Tumorformel: pT3, pN1a(ece-), L1, V1, Pn0.

Das Colon-ascendens-Karzinom wies eine distinkte Morphologie auf: Es fand sich ein scharf begrenzter, maximal die Muscularis propria infiltrierender rundlicher, stark muzinöser Tumorknoten, umsäumt und durchsetzt von einem dichten lymphozytären Infiltrat. Die schleimgefüllten zystischen Drüsen waren von einem meist einreihigen atypiearmen, becherzellreichen Zylinderepithel ausgekleidet. Entsprechende Tumorzellverbände flottierten frei in Muzinseen. Die Tumorformel für das muzinöse Adenokarzinom lautete: pT2, pN0, L0, V0, Pn0.

Immunhistochemisch zeigte sich in beiden Tumoren ein vollständiger Ausfall der Mismatch-Repair-Proteine MSH2 (Abb. 1e) und MSH6 bei erhaltener Expression von MLH1 und PMS2. Die molekulare Mutationsanalytik bestätigte eine hohe Mikrosatelliteninstabilität (MSI-H). Eine *BRAF*(V600E)-Mutation fand sich nicht.

Nebenbefundlich bot sich das Bild einer fokal gering aktiven Colitis ulcerosa mit Punctum maximum im Anorektalbereich, mit Kryptenumbau und fokaler Kryptendestruktion ohne Dysplasien (Abb. 1f).

## Wie lautet Ihre Diagnose?

Es handelt sich um ein Ileokoloproktokolektomiepräparat einer jungen Patientin mit zwei koinzidenten hochgradig mikrosatelliteninstabilen invasiven Adenokarzinomen (MSI-H), einmal vom konventionell-gemischten Typ und einmal vom muzinösen Typ, die auf dem Boden eines Lynch-Syndroms und gleichzeitiger Colitis ulcerosa entstanden.

 Das abschließende Tumorstadium lautete: pT3 (2), pN1a (1/31; ece-), L1, V1, Pn0, lokal R0.

## Verlauf

Im weiteren Verlauf ergab das bildmorphologische Staging mittels CT keine Hinweise auf Fernmetastasen. Die Patientin erhielt postoperativ über zweieinhalb Monate eine adjuvante Chemotherapie mit 6 Zyklen mFolfox (modifizierte Chemotherapie mit Folinsäure, 5‑Fluoruracil, Oxaliplatin). Stärkere Nebenwirkungen traten nicht auf. In der ersten Nachsorgeuntersuchung ergaben sich keine Hinweise auf ein Rezidiv. Eine humangenetische Beratung der Patientin wurde nach Abschluss der Chemotherapie initiiert. Parallel wurde die Wiedereingliederung der Patientin ins Berufsleben in Rücksprache mit dem Arbeitgeber veranlasst.

## Diskussion

Simultane kolorektale Karzinome (CRC) junger Patienten mit gleichzeitig bestehendem autosomal dominant vererbtem Lynch-Syndrom (LS, früher hereditäres nonpolypöses Kolonkarzinomsyndrom [HNPCC]) und Colitis ulcerosa (CU) sind selten in der Literatur beschrieben [1–4]. Beide Grunderkrankungen erhöhen voneinander unabhängig das Lebenszeitrisiko zur Entwicklung eines CRC, einerseits getriggert durch genetische Instabilität der chromosomalen DNA-Reparatur, andererseits durch einen chronisch-rezidivierenden Entzündungsprozess. Im Falle des Lynch-Syndroms können zudem Malignome anderer Organe auftreten, wie beispielsweise das hereditäre Endometrium- oder Ovarialkarzinom. 3 % aller neu diagnostizierten CRC liegt ein LS zugrunde; umgekehrt beträgt bei bekanntem LS das Lebenszeitrisiko für die Entstehung eines CRC je nach Literatur 6–77 % [5] oder 80 % [6], bei unüberwachten Patienten sogar 60–80 % [7]. Der Anteil mikrosatelliteninstabiler CRC an allen CRC wird auf 12–15 % geschätzt; hier sind allerdings auch die sporadisch auftretenden MSI-Tumoren miteingeschlossen [7]. Bei der CU liegt das CRC-Risiko 10 Jahre nach Diagnosestellung bei 1–2 %, mit ab diesem Zeitpunkt jährlichem Anstieg von 0,5–1,0 % [8, 9]. Das Lebenszeitrisiko für ein CRC bei kombiniertem Auftreten beider Grunderkrankungen ist laut einer großen niederländischen Studie von Derikx et al. [1] um 1,7 % gesteigert gegenüber der normalen Bevölkerung. Kolorektale Karzinome traten in diesem Kollektiv von insgesamt 1046 Fällen in signifikant jüngerem Alter auf (wie auch in unserem Falle einer erst 26-jährigen Patientin), mit einem mittleren Alter von 36 Jahren gegenüber einem mittleren Alter von 46 Jahren bei Patienten mit alleinigem Lynch-Syndrom. Das kumulative Risiko hingegen war in beiden Gruppen in etwa gleich (bei einer Grundlage von 15 Fällen mit kombiniertem Lynch-Syndrom und CED, hiervon 4 Fälle mit Entwicklung eines CRC [26,7 %], gegenüber 1031 Fällen mit alleinigem Lynch-Syndrom, hiervon 311 Fälle mit Entwicklung eines CRC [30,1 %]; [1]). In der zitierten Studie sowie in den Einzelfallbeschreibungen war das Auftreten eines CRC bei Lynchsyndrom und simultaner CED eher mit einer Colitis ulcerosa als mit einem Morbus Crohn assoziiert [1–5]. Molekular sind beide Erkrankungen unterschiedlich getrieben: Im Gegensatz zu der MMR-Defizienz beim Lynchsyndrom findet sich beim Kolonkarzinom auf dem Boden einer Colitis ulcerosa eine frühe *TP53*-Mutation. Diese folgt im Rahmen der chronischen Entzündung auf die Interaktion sogenannter reaktiver Sauerstoff- und Stickstoffspezies („reactive oxygen and nitrogen species“, RONS) mit der DNA und induziert Einzel- und Doppelstrangbrüche mit Veränderungen in codierenden und regulatorischen Sequenzen.

Typischerweise treten hereditäre rechtsseitige oder auch simultane Kolonkarzinome in vergleichsweise jungem Patientenalter auf, wie auch in unserem Falle. Wie De Jong et al. 2004 zeigten, spielt gerade bei MMR-defizienten Patienten die (rasche) Entstehung von CRC aus Adenomen eine bedeutende Rolle, was die jährlichen Vorsorgekoloskopien rechtfertigt [10], obwohl, wie unten aufgeführt, auch hiervon unabhängige Entstehungswege möglich sind. Histopathologisch können die hereditär bedingten CRC eine charakteristische, distinkte Morphologie aufweisen: Kennzeichnende histologische Typen sind das *muzinöse Karzinom*, das *medulläre Karzinom* und das *lymphozytenreiche Karzinom*; daneben deuten eine ausgeprägte intratumorale Heterogenität oder das Fehlen sogenannter „schmutziger“ Nekrosen auf eine hereditäre Genese hin [11]. Das eine unserer Karzinome war vom muzinösen Subtyp, mit einer typischen rundlichen Tumorform schüsselartig in der Darmwand und guter Abgrenzbarkeit zum umgebenden Gewebe. Das andere Karzinom wies ein heterogenes Wuchsmuster auf mit für kolorektale Karzinome ungewöhnlicher CK7- und Ca19.9-Expression und zeigte eine Lymph- und Hämangioinvasion und bereits eine Lymphknotenmetastase. Eine prominente lymphozytenreiche Immunantwort konnte hier nicht beobachtet werden. Dieses Karzinom entsprach nicht dem typischen Bild eines MSI-assoziierten Karzinoms und ähnelte Karzinomen, wie sie bei CED beschrieben sind, mit morphologischen und immunhistochemischen Aspekten eines pankreatobiliären Karzinoms.

**Diagnose:** Zwei koinzidente hochgradig mikrosatelliteninstabile invasive Adenokarzinome (MSI-H)

Molekularbiologisch liegt beim Lynch-Syndrom, dem häufigsten hereditären kolorektalen Karzinomsyndrom [5], eine Defizienz der Mismatch-Repair-Gene (MMR-Gene) vor, die zu einer Störung der DNA-Reparatur bei fehlerhaftem Basenaustausch führt.

Hiervon betroffen sind auch repetitive nichtcodierende, über das gesamte Genom hinweg verteilte DNA-Sequenzen („short tandem repeats“, sog. Mikrosatelliten). Der Nachweis einer Mikrosatelliteninstabilität ist ein molekularpathologisches Verfahren zur Diagnose einer MMR-Defizienz [9–11]. Am häufigsten finden sich Mutationen in den DNA-Reparatur-Genen *MSH2* und *MLH1*, seltener in *MSH6* und *PMS2*. Die vier MMR-Proteine formen einen Komplex aus je zwei Heterodimeren, der fehlerhaft in die DNA eingebaute Basen detektieren und ersetzen kann. Hierbei benötigen MSH6- das MSH2-Protein und PMS2- das MLH1-Protein als stabilisierenden Partner, ohne diesen werden sie ubiquitiniert und proteosomal degradiert. Als einfache Screeningmethode genügt somit die alleinige immunhistochemische Prüfung auf einen Ausfall von MSH6 und PMS2, um eine Aussage über ein Funktionsdefizit aller vier Proteine zu erhalten.

Von der *MLH1*-Mutation ist die funktionelle epigenetische Stilllegung des *MLH1*-Gens durch eine meist sporadische Hypermethylierung des MLH1-Promotors abzugrenzen.

Letztere tritt insbesondere bei älteren Nicht-Lynch-Patientinnen mit rechtseitigem Kolonkarzinom auf. Diese lässt sich in den von einer primären *BRAF*(V600E)-Mutation getriebenen, sessilen serratierten Läsionen mit Dysplasie (SSL-D) beobachten. SSL‑D sind direkte Vorstufen des sporadischen *BRAF*-mutierten, mikrosatelliteninstabilen Kolonkarzinoms.

Patienten mit *MSH2*- oder *MLH1*-Mutationen entwickeln Karzinome häufiger in jüngerem Alter als Patienten mit *MSH6*- oder *PMS2*-Mutationen [12]. Selbst häufige Kontrollkoloskopien bei bekannten Lynch-Patienten in einem Abstand von ein bis zwei Jahren [13] können die Karzinomentstehung in diesen Zeitspannen nicht verhindern, wie Argillander et al. 2018 in einer prospektiven Analyse an einem niederländischen Kollektiv zeigten. Hierfür verantwortlich sind die Biologie dieser Tumoren mit einer raschen Adenom-Karzinom-Sequenz sowie das häufige Vorliegen flacher Adenome, die koloskopisch schwer zu detektieren sind [14–17]. Diese „flachen“ Vorläuferläsionen mit Dysplasien stellen auch bei der CU eine diagnostische Herausforderung dar. Sie sind endoskopisch schwer zu erkennen und bei bioptischem Zufallsbefund einer Dysplasie auch im Hinblick auf eine komplette endomukosale Abtragung kaum wiederzufinden. Weiter postulierten Ahadova et al. 2016 die These eines nichtpolypösen Karzinogeneseweges, der den sogenannten „Intervall-Krebs“ miterklären könnte: Sie detektierten *CTNNB1*-Mutationen in Lynch-Syndrom-assoziierten Kolonkarzinomen, die offenbar direkt invasiv wuchsen, ohne den vorherigen Zwischenschritt einer fakultativen adenomatösen Präkanzerose zu nehmen und damit die Chance einer frühzeitigen Detektion reduzieren [18].

Das hereditäre mikrosatelliteninstabile Kolonkarzinom weist im Vergleich zum konventionellen sporadischen Kolonkarzinom eine gute Prognose auf, solange es nicht lymphonodal metastasiert ist. Aus den zahlreichen Mutationen im Tumor resultieren fehlgefaltete Proteine, sogenannte „Frameshift-Polypeptide“. Neoantigene provozieren eine ausgeprägte T‑Zell-dominierte Immunreaktion gegen den Tumor, unter Induktion eines proinflammatorischen, zytokinreichen Milieus. Insbesondere CD8^+^-T-Lymphozyten scheint hier als sogenannten „tumour infiltrating lymphocytes“ (TILs) eine entscheidende Bedeutung zuzukommen. Sie induzieren eine Frameshift-Polypeptid-spezifische Immunantwort [19–21], die das Karzinom selbst bis in ein histopathologisches pT4-Stadium lange in Schach halten kann.

Sobald es aber den Tumorzellen gelingt, durch „Selektionsdruck“ bestimmte Veränderungen zu durchlaufen, die es ihnen ermöglichen, der Immunantwort zu entfliehen, liegt eine vergleichsweise äußerst ungünstige Prognose vor [22]. So zeigen sich bei mit Lymphknotenmetastasen nicht mehr so ausgeprägte Immunreaktionen, wie sie in frühen Stadien zu beobachten sind [22, 23], was auch wir in unserem Falle sowohl eines fortgeschrittenen, lymphonodal metastasierten Karzinoms mit kaum begleitender Immunreaktion und als auch eines simultanen Karzinoms im frühen Stadium mit ausgeprägter umgebender Immunantwort dokumentieren konnten.

## Ausblick

Inwieweit das proinflammatorische Milieu *bei Koinzidenz* von Lynch-Syndrom und chronisch entzündlicher Darmerkrankung die *stadienabhängige* Entwicklung von Krebsvorläufern und manifesten kolorektalen Karzinomen beeinflusst, ist eine offene Frage. Dies berührt auch die Therapie nichtresektabler Karzinome: So scheint die 5‑FU-Therapie bei irresektablen MMR-defizienten CRC kein gutes Ansprechen zu zeigen; hier ist unabhängig vom immunhistochemischen Expressionsstatus eine PD-L1-Inhibitor-Therapie z. B. mit Pembrolizumab indiziert mit guten Ansprechraten [9–11, 24–29]. Im Falle eines metastasierten Karzinoms kann Nivolumab verabreicht werden. Hier wäre zu überprüfen, ob dieses Therapiekonzept auch bei koinzidentem Lynch-Syndrom und chronisch entzündlicher Darmerkrankung trägt.

## Fazit für die Praxis

- Die seltene Kombination eines hereditären Lynch-Syndroms mit einer Colitis ulcerosa lässt ein nochmals erhöhtes Risiko für die Entwicklung eines kolorektalen Karzinoms in ungewöhnlich jungem Lebensalter vermuten, bedingt durch zwei unterschiedliche Wege der Karzinogenese und ein defektes lokales Immunsystem, das möglicherweise nur bedingt in der Lage ist, Krebsvorstufen und invasive Karzinome in Schach zu halten.
- Beide Erkrankungen können endoskopisch schwer zu detektierende flache dysplastische Vorläuferläsionen aufweisen, aus denen rasch invasive Adenokarzinome entstehen können. Zudem ist beim Lynch-Syndrom die Adenom-Karzinom-Sequenz beschleunigt, mit einem erhöhten Risiko für einen sog. „Intervall-Krebs“ trotz vollständiger und gänzlich einsehbarer Koloskopie. Daher ist eine engmaschige Kontrolle dieser Patienten mit vollständiger Koloskopie anzuraten.

## Abkürzungen

CA19.9: Carboanhydrate-Antigen 19.9
CED: Chronisch entzündliche Darmerkrankung
CK7: Zytokeratin 7
CRC: Kolorektales Karzinom
CU: Colitis ulcerosa
DNA: Desoxyribonukleinsäure
HE: Hämatoxylin-Eosin
HNPCC: Hereditäres nonpolypöses Kolonkarzinomsyndrom
LS: Lynch-Syndrom
mFOLFOX: Modifizierte Chemotherapie mit 6 Zyklen Folinsäure, 5‑Fluoruracil, Oxaliplatin
MLH1: MutL homolog 1
MMR: Mismatch-Repair
MSH2: MutS homolog 2
MSH6: MutS homolog 6
MSI: Mikrosatelliteninstabilität
PAS: Periodisches Schiff-Reagenz
PMS2: Post-meiotic segregation increased 2
SSL‑D: Sessile serratierte Läsion mit Dysplasie
TILs: Tumour infiltrating lymphocytes
